# Enhancing healthcare providers’ diagnostic and intervention skills to deal with suicidal patients at emergency departments in the Palestinian hospitals: a quasi experimental study

**DOI:** 10.1186/s12913-023-10256-6

**Published:** 2023-11-06

**Authors:** Samah Jabr, Fayez Mahamid, Zaynab Hinnawi, Dana Bdier

**Affiliations:** 1 Community Mental Health Center, Palestinian Ministry of Health, Ramallah, Palestine; 2 Psychology and Counseling Dept, An-Najah National University, Nablus, Palestine; 3 University of Milano-Bicocca, Milan, Italy

**Keywords:** Health care providers, Suicide intervention, Emergency department, Palestine

## Abstract

**Background:**

Suicide cases in Palestine continue to record a remarkable annual increase, but we lack a comprehensive verified national data collection system of suicide and it is expected that real numbers of attempted/suicide in Palestine are higher because not all suicide or attempted suicide cases are reported. The purpose of this study was to test the effectiveness of a time-limited training intervention in enhancing healthcare providers’ diagnostic and intervention skills to deal with suicidal patients who visit emergency departments in Palestinian hospitals.

**Methods:**

The sample consisted of 43 healthcare providers who work in public hospitals in the northern of the West Bank of Palestine, ranging from 25 to 56 years, involving 36 males and 7 females. A one-group quasi-experimental design was used, in which the experimental group received a training program to enhance healthcare providers’ diagnostic and intervention skills to deal with suicidal ideations and attempts, the intervention lasted for 8 weeks, with 1 session per week. The performance of the experimental group was tested before and after the intervention.

**Results:**

Our findings revealed the effectiveness of the training intervention in enhancing participants’ suicide assessment, diagnosis, and self-care skills.

**Conclusions:**

These results suggest that a brief and carefully developed training intervention can potentially change healthcare providers’ perceptions and behaviors toward suicide with a possible impact on clinical care therein.

## Theoretical background

Like other Arab countries, studies about suicide and attempted suicide in Palestine are limited, therefore statistics are rare; this is suggested because of that reporting does not precisely reflect suicide incidences, because of stigma and religions which all prohibit suicide, with Islam having the most forbidding attitude towards it [22]. The Quran emphasizes the sanctity of human life, underscoring that it cannot be terminated without valid justification. The right to life is an inherent principle in Islam, recognizing life as a divine gift from the Creator, which carries the obligation to preserve it. Suicide motivated by despair in God’s mercy or due to worldly issues is unequivocally prohibited [15].

Twelve countries with Muslim Majority were included in a study with university students conducted by Eskin and colleagues, indicated that suicidal thoughts and attempts were quite frequent among young adults. It showed that number of students who had attempted to take their lives by suicide was the second highest in Palestine following Saudi Arabia (38.7% − 23.6%) in comparison with other Muslim -majority countries [8]. Another study in which a school-based survey was submitted to students aged 13–15 in Gaza and West Bank, with the purpose of exploring suicidal ideation, revealed that the overall prevalence of suicidal ideation and/or planning was 25.6%, and it was higher than the rates of other countries in the Eastern Mediterranean Region [20]. Statistics are more critical in Gaza with mental health becoming endemic there, an estimated 38% of young people have considered suicide at least once [19]. Another study conducted by [10], which is a descriptive cross-sectional one, aimed to ascertain the prevalence of mental health-related behaviors among 720 Palestinian school adolescents in Tarqumia. The results revealed a high prevalence of suicide ideation (24.58%) and suicide attempts (25.28%) among Palestinian adolescents, with a higher risk observed in males. Additionally, a substantial portion (25.28%) of both male and female adolescents reported feelings of loneliness. These findings underscore the urgent need for intervention strategies to address the rising trend of suicide ideation among vulnerable populations in Palestine, and this study may provide valuable insights for the development of preventive measures.

A cross-sectional study explored suicidal ideation and attempts among Palestinian students exposed to prolonged political and domestic violence. 303 college and university students, aged 18 to 23, completed anonymous self-report questionnaires assessing suicidal ideation, posttraumatic stress symptoms (PTSS), depression, anxiety, and sleep problems over the past year the study conducted in. The results revealed a high prevalence of suicidal ideation and attempts, as well as elevated levels of PTSS, depression, and sleep problems compared to other college samples. Path analysis demonstrated that PTSS had a direct association with suicidal ideation and an indirect one through its connection with sleep problems and depressive symptoms. These findings emphasized the mental health challenges faced by students enduring extended exposure to violence, underscoring the importance of implementing interventions to reduce depression and PTSS for healthcare and academic institutions as a critical step in suicide prevention among college students exposed to trauma [14].

Suicide is difficult to address in Palestine because it is considered as a crime, a sin, and it can occasionally be disguised as a nationalistic confrontation with the occupation. Article 339 of Law No. 16 of 1960 in the Palestinian Penal Code states that “Inducing a person to commit suicide or aiding them in doing so, using any of the methods mentioned in Article 80, is punishable by temporary detention. If the suicide attempt is ongoing, the individual will be sentenced to imprisonment for a period ranging from three months to two years. The punishment can extend up to three years if it results in harm or permanent disability” [7].

Based on practioners observations, it’s uncommon for Palestinian patients and their families to openly disclose personal or family histories of suicidal tendencies when directly questioned during clinical assessments. Even when a strong therapeutic bond has been established, many individuals tend to deny symptoms associated with suicidality. While conservative Muslim and Christian religious beliefs may offer protective factors against suicide within the Palestinian population, concerns about the stigma associated with suicide can hinder effective treatment [21].

In numerous instances where medical professionals identify cases as suicide, families often attempt to disprove this diagnosis and exert pressure on healthcare personnel to avoid officially documenting it as such due to concerns about its impact on the family’s reputation within the community [26].

Thus in countries where attempting suicide is counted illegal, and where suicide still a taboo and the stigma around it is very high, reports of incidents of unnatural death such as drug overdoses or car accidents are likely to increase [31]. The Palestinian Police Research and Planning Department offers some statistics about suicide in Palestine (35 deaths and 218 attempts in 2018), along with the suggestion that these numbers are not precise, as the majority of cases are not reported [25].

Usually, people with suicidal attempt don’t go to the public hospitals for treatment, they are not treated at all or treated at their homes or in private clinics and hospitals [22]. And the fact that they reach the public hospitals, does not mean they will be reported as suicidal attempts, because they may go unidentified [17]. Further, regardless their chief complaint, approximately 10% of all adult emergency department patients have recent suicidal behavior or ideation, but unless being asked, many will not disclose [2, 5, 18].

Common misconceptions, such as the belief that patients who reveal suicidal thoughts are unlikely to act on them, were widespread, and GPs who felt uneasy or underprepared to discuss suicide with patients typically showed reduced involvement in assessing suicide risk [4] .In fact that Medical schools often lack proper education on the dynamics of suicide prevention [29], Practitioners’ avoidance is related to their underlying fear of the belief that talking about suicide will let people consider or follow coping behaviors which result in suicide [21], and also because of the contradictions brought to the therapeutic setting by suicidal patients [29]. Furthermore, healthcare providers are unclear as to where to refer patients, despite having National Mental Health Referral Guidelines [21].

Staff of emergency departments in Palestinian hospitals are reported to have negative and ambivalent attitude toward suicidal patients (Doctors engage less in the treatment unless there is a serious physical injury). These patients are exposed to lack of empathy and stigmatization. Accordingly, quality of care offered to these Patients is decreased, and an important opportunity to prevent repetitive self-harming behavior is missed. That’s why there is a need for protocols, proper guidelines and education for the emergency staff [29]. Few of the staff thought treatment of suicidal patients is almost always a top priority to them [3], so they should struggle to conquer their area of discomfort [2].

A study conducted by Médecins du Monde Switzerland to assess care provided to people who attempts suicide in four hospitals in West Bank, had many interesting results [25]. They found that it is emergency room doctors who handle suicidal attempts, are the ones who decide whether that individual should be admitted to a psychiatric hospital, referred to Community Mental Health Center for follow-up, or stay in the hospital. Although patients’ observations about emergency units’ healthcare services were generally positive, they announced receiving deficient information in regard to treatment outline, follow-up care, benefits and risks of treatment. They also complained about some health staff judgmental and negative attitudes. Only half of the patients had had an organized follow-up appointment with a psychiatrist. Emergency department staff were found to lack skills and expertise in regard to case evaluation of individuals who had attempted suicide, treatment, and referral, and they showed their need for more specialized training.

Certain patients held the belief that the limited financial resources available in government hospitals hindered the ability to undertake appropriate measures [25], although Ministry of Health (MoH) provides psychological treatment after discharge for free, as part of The National Committee of Suicide Prevention actions to prevent suicide. Other actions have included building capacity of nurses and doctors, in particular, on suicide intervention. Further, The National Suicide Prevention Strategy 2021–2026 has many objectives including: improving case reporting in hospitals, reducing stigma and taboo and increasing awareness about self-harm and suicide among health service providers. Ensuring early detection on the level of primary health care is a central element in suicide prevention, therefor, training professionals to detect suicide ideation, risk factors, suicide attempts, and self-harm behaviors ,is a crucial factor in enhancing services’ capacity [21].

In the light of results of previously mentioned study conducted by MDM, the research team advised for some recommendations; increasing skills, knowledge and awareness of healthcare providers about suicide, training professionals how to support family members and to include them in detection and management of any member at risk, more focused post-care must be provided by care providers, increasing in-depth understanding of risk factors such as previous attempts, mental illness, family conflict, financial crisis, and martial problems [25]. Besides, raising awareness about controlling access to means of suicide such as poison and pesticides is another element in suicide prevention [30].

Emergency departments (ED) are important venue for suicide prevention actions to take place for many reasons; ED screen regularly for health concerns that are outside the complaint patients presented with [16]. As well as, ED patients are suggested to be vulnerable, having a higher risk of developing psychiatric complications [12, 13], and screening populations with high risk is more efficient [6]. In addition, ED staff could offer a unique opportunity for suicidal patients to intervene or refer to specialized resources [1, 11, 12]. Efforts of screening implementation in the ED can identify patients with high risk who may not receive care in other settings [18].

Recent systemic review for studies about training interventions for emergency department staff and psychosocial interventions delivered by ED staff for people who self-harm was conducted, including fifteen studies. The review indicated improvement in knowledge and attitudes of emergency staff, and betterment in skills of assessment and providing companionate care for suicidal patients. It also showed that some interventions (like safety planning and follow-up contacts) can be delivered by ED staff and are linked to decrease in repeat suicide attempts [35].

Due to the lack of previous studies designed to test the effectiveness of interventions in improving healthcare providers’ diagnostic and intervention skills to deal with suicidal patients in the Palestinian context, the current study was conducted to explore the effectiveness of a time limited training program to improve diagnostic and intervention skills among Palestinian health care providers. The current study hypothesized that the training program would improve the healthcare providers’ diagnostic and intervention skills to deal with suicidal patients who visit emergency departments in Palestinian hospitals?

## Methods

### Participants and procedures

The study was conducted in October (2022) and targeted [43] health care providers out of 150 health care providers working at emergency departments in five Palestinian Governmental hospitals in northern West Bank (Rafidia hospital in Nablus, Yaser Arafat hospital in Salfit, Medical Palestinian Complex in Ramallah, Thabet Thabet hospital in Tulkarem and Darweesh Nazzal hospital in Qalqilyah). The sample was chosen using simple random sampling form those who agreed to participate in the study. Participants were informed about the purpose of the research and provided with information that allowed them to decide whether or not they wished to continue participating in the study. Consent was verbally obtained before the training took place. The age range was between (25–56) years, involving 36 males and 7 females. For inclusion in the study, participants were required to be: (1) Licensed psychologists, clinical social workers and physicians, (2) native Arabic speakers, and (3) living in the West Bank of Palestine. The performance of the [43] health providers was tested before and after the intervention using a quasi-one group experimental design, as it was the most appropriate design to be used in our study. The training program lasted for two months, and included a set of competencies, skills and knowledge that may help health providers to deal with suicide attempt cases. Our study was approved by An-Najah institutional review Board (IRB) before data collection was initiated.

### Study instruments

#### Suicide assessment skills scale

The suicide assessment skills scale consisted of 20 items designed to test suicide assessment skills among health providers. The authors developed the scale based on the practical and theoretical literature in this filed [6, 23, 25, 31]. Participants responded to items of the scale on a 5 -point Likert scale ranging from 5 (very high) to 1 (very low). Item scores were combined into a sum score, with higher scores indicating higher suicide assessment skills. A committee of experts totaling [10] reviewed the items of the scale for content validity and comprehensiveness. (80%) as a percentage of agreement between experts was used for each item. Accordingly, the researchers changed the interpretation for some items, on the basis of feedback from the committee members. We also test the construct validity of the scale among a sample of [80] physicians (the validation sample), The scale indicated a high level of construct validity in assessing suicide assessment skills and correlations between items, with the total score of the scale ranging between (0.40 − 0.57). Moreover, the results of an exploratory factor analysis (EFA) indicated a stable one factor construct of our scale. Finally, Cronbach’s alpha coefficients indicated high internal consistency for the total scale (0.92).

#### Suicide intervention and self-care skills

The suicide intervention and self-care scale designed to test suicide intervention, knowledge and self-care skills of health providers. The authors developed the scale based on the practical and theoretical literature in this filed [6, 24, 31]. The scale ended with 20 items represented by three sub-scales; Knowledge which includes 9 items [1, 2, 4, 5, 8, 31, 10, 12, and 14], skills sub-scale, which also included 5 items [3, 6, 13, 15, and 16], and self-care subscale also includes 6 items [7, 11, 17–19, and 20]. For the 20 items of the scale, a positive response is scored as a 1 (yes) and a negative response as 0 (no) Item scores were combined into a sum score, with higher scores indicating higher suicide intervention and self-care skills. A committee of experts totaling [10] reviewed the items of the scale for content validity and comprehensiveness. (80%) as a percentage of agreement between experts was used for each item. Accordingly, the researchers changed the interpretation for some items, on the basis of feedback from the committee members. We also test Exploratory factor analysis (EFA), and Confirmatory factor analysis (CFA) among a sample of [80] physicians (the validity sample), the results revealed a three stable construct of the scale in assessing suicide intervention and self-care skills among physicians. Cronbach’s alpha coefficients of the scale indicated a high internal consistency for the total Instrument (α = 0.92), and for the subscales; Knowledge (α = 0.90), self-care (α = 0.88), and self-care (α = 0.91).

### Intervention program

The training aimed to provide ED teams with a set of competencies, skills and knowledge for dealing with suicide attempt cases and their families, thus enhancing the detection and management of at-risk patients arriving at the ER, as well as providing proper referrals to out-patient care services. It also aimed to empower ED teams to have a positive attitude and develop therapeutic alliance with patients to prevent the risk for relapse after discharge by developing a safety plan for each patient and providing safe outpatient referral pathways. Moreover, the program intended to educate workers in ED on self-care and psychological well-being and how to manage stress and burnout. In addition, it is designed to help healthcare professionals in ED in continuing to build their capacity to provide effective care to patients at risk of suicide.

### Description of the program

The training program (Table 1) included 30 h of training for each group/ hospital. 20 h were designated for the topic of “Soft Skills in Dealing with Suicide Patients at ED and their Families,” and 10 h for “Self-care and Stress-management for ED Staff.” A manual was prepared ahead of the training, reviewed and endorsed by the Mental Health Unit (MHU) at the MoH, to guide the training program. The training included important definitions related to suicide, a global context on suicide through the presentation of international studies and statistics, as well as the local context for suicide in Palestine. It also focused of suicide among adolescents and youngsters and how to interview them. The training presented official numbers recorded for attempted/suicide in Palestine, as well as the findings of the few local studies on the subject. A discussion on the discrepancy between official numbers registered with the police and other institutions took place emphasizing a need for a proper national reporting mechanism of attempted/ suicide cases in Palestine. The training included many case discussions, videos, group activities and brainstorming exercises.

**Table 1:** Training-program sessions

*Sessions*	*Strategies and topics*
1st Session Relationship Building	Group introductions Inform members about the process Share expectations with the group Determine personal aims related to the process Determine rules for the group Assessment and summary of session
2nd session	Definitions of suicidal items Significance of the training The role emergency workers can play The suicide treatment gap Examination of health providers views on suicide Societal stigma and taboo Understanding the factors leading to suicide Protective factors and gender considerations regarding suicide
3rd session	Risk of suicide screening Primary and secondary tests Suicide risk assessment Risk levels and intervention Treatment and referral decisions The necessary skills how the examination is done and lifeguard map
4rd session	Maintaining patient safety Prevention interventions in emergency departments Psychoeducation, safety planning Lethal means limitation Quick referral Aftercare contacts Safe patient discharge process
5th session	Suicide and special age considerations Parent/family/caregiver involvement in treatment Suicidal infection Suicide among children and adolescents Youth suicide prevention Professionals’ response to suicide (immediate action management, emotional response management) Suicide case review, and professional growth and development
6th session	Psychological needs of emergency staff Stress, vulnerability, resilience, burnout, and medical mistakes, Secondary trauma experiences. Second victim syndrome. Physician’s ill-fitness to work.
7th session	Concept of the importance of face to face communication in in social aspects Gain skills in effective listening and dialogue Develop skills to communicate effectively with friends and teachers Avoid social isolation and addictive behaviours Assessment and summary of session
7th session	Working within a team Staying safe at work Mindfulness Debriefing Impact of shift work on public health Documentation Supervision.
8th session Assessment	Summary of sessions; reviewing experiences during the process Members share assessment of personal development and group development during the process Ensure positive emotions are felt at the end of the process Final activity and appropriate finish to the group

To shift attitude towards suicide patients, participants underwent an exercise that includes a set of questions to examine their preconceived notions and attitudes towards suicide. Through this exercise, participants were able to think about their personal biases on the topic of suicide without having to share their answers. The trainer’s intervention was to explain and emphasize that self-awareness about their own opinions and attitudes about suicide can help them improve interaction with patients and thus develop the care that they provide to these patients. Likewise, a negative attitude towards suicide may cause missed opportunities to save a life or offer hope and assistance to patients in-need. It may also lead to an exaggeration in reactions when patients are identified as suicide attempters. There was also a lengthy discussion on the set of general ideas related to linking suicide to religious misconceptions and traditional societal values, as well as their impact on the provision of services and care to patients. A roadmap for service provision at the ER for attempted suicide patients was developed for this training, it includes the stages of identifying patients at risk, levels of risk, and the required action by designated ED staff member. Two of the co-authors of this manuscript (a clinical psychologist and a psychiatrist) implemented the intervention program, and both experts are licensed by Palestinian Ministry of Health as professionals in suicide interventions. A Committee of 10 experts (clinical psychologists, psychiatrists, social workers) reviewed and approved the intervention protocol.

### Statistical analysis

The data of our study was normally distributed; therefore, we used parametrical tests, in which means and standard deviations were calculated for all items of the scale on pre and post assessments, while independent samples t-test was calculated to evaluate the differences between males and females on pre and post assessments. Moreover, paired sampled t-test was performed to test the significance of differences between pre and post assesments among the experimental group. Finally, EFA, CFA, Person Correlaion’s Cofficient, and Chronbakch Alpha Forumala were used to test the psychomatic propaties of the suicide assessment skills, and suicide intervention and self-care skills scales.

## Findings

Table 2 indicated that participants obtained low scores on pre-assessment in regard to suicide assessment skills, while participants recorded very high scores on post-assessment.

**Table 2:** Means and standard deviations on pre and post assessments suicide assessment skills (N = 43)

No.	Items	Pre-assessment		Post-assessment	
		M	SD	M	SD
Q1	I have sufficient knowledge of the early indications of the possibility of suicide	2.60	0.84	4.51	0.50
Q2	I know the role of the emergency department health care provider in suicide prevention	2.94	1.02	4.34	0.48
Q3	I have knowledge of the personal, social and societal factors affecting the occurrence of suicide	3.05	1.08	4.14	0.60
Q4	I have knowledge of protective factors that prevent relapse in people who have committed suicide	2.37	0.843	3.91	0.70
Q5	I know the warning signs of imminent risk of suicide	2.51	0.95	4.28	0.89
Q6	I can use assessment tools to screen for suicide	2.37	0.97	4.11	0.90
Q7	I have sufficient knowledge of the methods of attempts to conceal suicide	2.65	0.90	3.97	0.70
Q8	I have the skills to work with people who have committed suicide	2.71	1.01	3.97	0.56
Q9	I have knowledge of the skills and interventions that can be used to prevent suicide while the patient is in the emergency department	2.82	0.82	4.20	0.47
Q10	I can build and develop a safety plan for the person who has attempted suicide	2.60	0.91	4.40	0.60
Q11	I have psychoeducation skill for patients with risk of suicide	2.80	1.13	4.37	0.54
Q12	I know what is meant by limitation of lethal means and how to negotiate it with the patient	2.60	0.84	4.48	0.50
Q13	I know how to do fast referral with its different steps	2.42	1.00	4.57	0.69
Q14	I am skilled in designing a discharge plan for the patient who has attempted suicide to improve his transition from the hospital to the community	2.45	1.06	4.60	0.60
Q15	I know the specificity of suicide among children and adolescents	2.62	1.03	4.42	0.65
Q16	I know enough about the phenomenon of suicidal infection and how to reduce it	2.65	0.83	4.45	0.50
Q17	If a patient with whom I have dealt has committed suicide, I am skilled enough to respond to the event in a way that preserves my psychological well-being and my role as a professional	3.17	1.04	4.68	0.63
Q18	I am fully aware of the psychological impact of occupational pressures on groups that work in the front lines in the face of danger, such as emergency crews	3.34	1.08	4.60	0.55
Q19	I have the ability to verbally de-escalate the agitated person in front of me	3.14	0.845	4.42	0.55
Q20	I can do a debriefing after every unusual or negative event	2.91	0.919	4.54	0.56
Total		2.74	0.631	4.35	0.27

Results of Table 3 showed no significant differences between males and females on post-assessment of suicide assessment skills, which indicates the effectiveness of the intervention program in improving suicide assessment skills for all participants.

**Table 3:** Differences between males and females in suicide assessment skills (N = 43)

Post-assessment	variable	M	S.D	T .value	DF	Sig
suicide assessment skills	males	4.34	0.28	0.06	31	0.94
	Females	4.33	0.25			

**Fig. 1:**
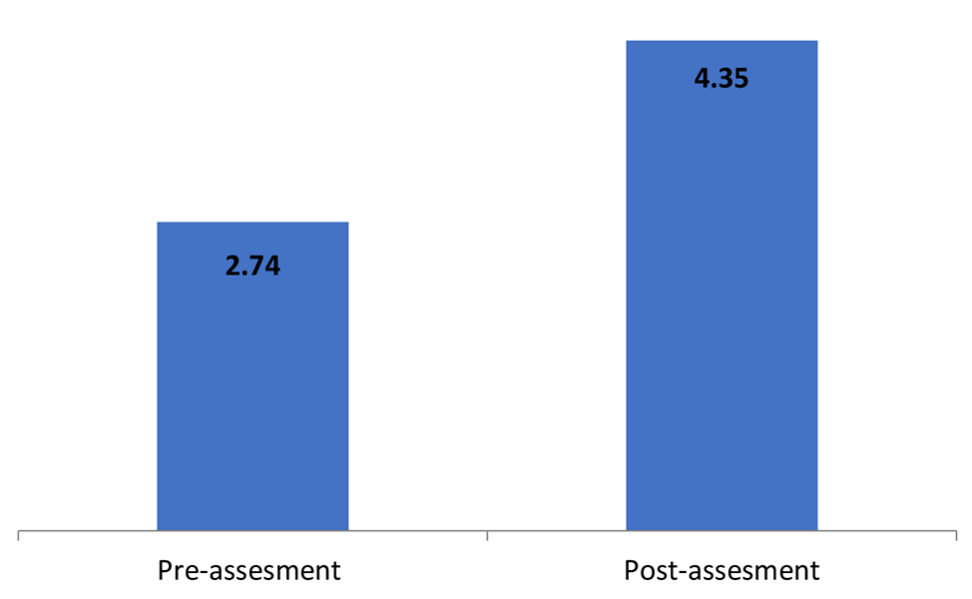
Suicide assessment skills on pre and post-assessments

Results of Table 4; Fig. 1 showed significant differences in suicide assessment skills between pre-assessment and post –assessment in favor of post-assessment, which indicating the effectiveness of the intervention program in improving suicide assessment skills among health providers.

**Table 4:** Differences between pre and post assessments in suicide assessment skills (N = 43)

Pre & post assessments	variable	M	S.D	T .value	DF	Sig
Suicide assessment skills	Pre-assessment	2.74	0.28	-13.20	34	*0.000
	Post-assessment	4.35	0.25			

Results of Table 5 showed no significant differences between males and females on post-assessment of suicide intervention and self-care skills, which indicates the effectiveness of the intervention program for all participants.

**Table 5:** Differences between males and females in suicide intervention and self-care skills (N = 43)

Post-assessment	variable	M	S.D	T .value	DF	Sig
Knowledge	males	0.88	0.09	− 0.44	31	0.65
	Females	0.90	0.10			
Skills	males	0.7304	0.22	− 0.49	31	0.62
	Females	0.7667	0.16			
Self-care	males	0.6739	0.30	0.81	31	0.42
	Females	0.5833	0.32			
Total score	males	0.8043	0.11	0.04	31	0.99
	Females	0.8042	0.13			

**Fig. 2:**
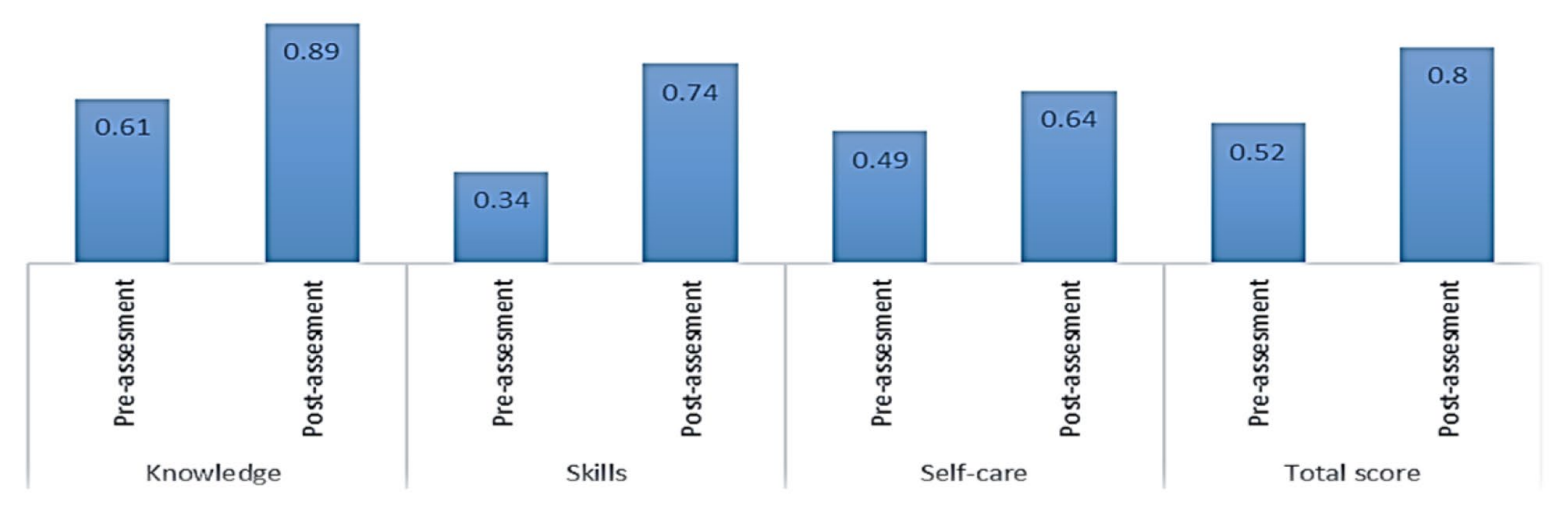
Suicide intervention and self-care skills on pre and post assessments

Results of Table 6; Fig. 2 showed significant differences in suicide intervention and self-care skills between pre-assessment and post-assessment in favor of post-assessment, which indicating the effectiveness of the intervention program.

**Table 6:** Differences between pre and post assessments in suicide intervention and self-care skills (N = 43)

Pre-assessment / post-assessment	variable	M	S.D	T .value	DF	Sig
Knowledge	Pre-assessment	0.61	0.12	-10.552	33	*0.000
	Post-assessment	0.89	0.10			
Skills	Pre-assessment	0.34	0.18	-9.526	33	*0.000
	Post-assessment	0.74	0.20			
Self-care	Pre-assessment	0.49	0.28	-2.433	33	*0.000
	Post-assessment	0.64	0.31			
Total score	Pre-assessment	0.52	0.11	-11.901	33	*0.000
	Post-assessment	0.80	0.12			

## Discussion

To our knowledge, this is the first study to examine the effectiveness of training interventions for health care providers who work with suicidal patients at emergency departments in Palestinian public hospitals. The findings of our study indicated the effectiveness that the training program in improving participant’s suicide assessment skills. Moreover, results showed the effectiveness of the training intervention in improving participants’ self-care, knowledge, and intervention skills. Our results are consistent with previous findings indicated the effectiveness of training programs in enhancing intervention skills of health providers to deal with suicidal attempts and ideations, for example [28] tested the effect of clinical training in improving mental health professionals’ skills to assess and manage suicidal behaviors. Results indicated that 44% of practitioners reported increased confidence in assessing suicide risk, 54% reported increased confidence in managing suicidal patients, 83% reported changing suicide care practices, and 66% reported changing clinic policy [9]. evaluated the effectiveness of training course for ‘‘front-line’’ clinical staff in the recognition, assessment and management of suicide risk. Results showed that the training course improved in the skills and confidence of the trainees which were sustained at the two month follow up [32] evaluated the impact of training program in improving suicide assessment, management, and treatment of health care professionals. Results from pretest and posttest showed improvements in knowledge and attitudes about suicide and confidence in treating at-risk individuals.

One possible explanation for our result is that the training program included several techniques, theoretical and practical tasks that enhanced health care providers’ knowledge, self-care, and suicide assessment skills. Our training program included different concepts and definitions related to suicide, a global context on suicide as well as the local context for suicide in Palestine. It also focused on how to deal with suicidal patients among clinical and non-clinical groups, especially, suicide among adolescents and youngsters and how to interview them.

Enhancing Palestinian health care providers’ skills to deal with suicidal patients is crucial in Palestine, as the situation in the occupied territories of Palestine is fraught with environmental stressors (militarization, poverty, lack of employment opportunities, cultural pressures, etc.) and few positive social outlets due to the restrictions on movement between communities, a lack of recreational facilities, and cultural standards of gender separation. In this situation, the vulnerability to suicide attempts due to prolonged and traumatic experiences increases [27, 33]. In a study aimed to determine the prevalence of suicide ideation and attempt among Palestinian adolescents, with results a high prevalence rate (24.58%) of suicide ideation and suicide attempt (25.28%) among Palestinian adolescents was noted.

Another important topic which was part of this training and has a critical priority was patient safety management in Palestinian hospitals. A scoping review that included twelve studies, conducted by [34] to review training interventions targeted health workers on inpatient suicide prevention, showed that suicide prevention interventions had positive effect patient safety management, skills and attitude of health care providers in public hospitals. In addition to the post-test results, there was important feedback from the participants indicating how valuable this training was in expanding their knowledge about suicide intervention and changing their attitudes towards suicide. A psychologist admitted that he felt these patients were manipulative and undeserving of medical attention. He acknowledged that his attitude changed drastically after the training. Another participant said that he had difficulty dealing with mental health patients; their conditions are ambiguous and have no place in the emergency department. He changed his mind after the training pointing to the importance of the availability of a road map and simple steps to make things clearer for himself and his colleague.

Our findings revealed that there was no significant difference in suicide assessment skills when gender was taken into account, and this result could be explained as male and female healthcare providers received the same theoretical and practical training, and they also work in the same places, so they are affected by each other’s attitude and practices. The results showed that both male and female healthcare providers had similar improvements in post-assessment scores, demonstrating the effectiveness of the training program in enhancing the intervention skills of healthcare providers to deal with suicidal patients.

The current study has several limitations that may offer opportunities for future research. First, our study targeted Palestinian health care providers who work in public hospitals in northern West Bank of Palestine. More studies targeting Palestinian healthcare providers in private and public hospitals in different areas of the West Bank of Palestine are needed to generalize the findings. Second, the study used only one group experiment design, future studies using two groups (experimental and control) design are needed to establish a cause-and-effect relationship by isolating the effect of an independent variable. Third, the training intervention used in this study was not tested before in the Palestinian context, and therefore, more studies are needed to prove the efficacy of the training program to enhance health care provider’s skills to deal with suicidal ideations and attempts.

## Conclusion

The current study was designed to test the effectiveness of a time limited training program in improving health care providers’ skills to deal with suicidal attempts and ideation among patients who visit emergency departments in Palestinian public hospitals. The findings or our study revealed the effectiveness of the training intervention in enhancing health care providers’ skills to deal with suicidal ideations and attempts. Overall, the results of this study showed that a brief and professional training on clinical care for suicidal patients can increase health provider confidence and favorably impact the clinical practice of practitioners. These findings support conducting future studies targeting health and mental health providers to enhance their skills in developing individual and group therapeutic interventions to deal with suicidal patients in situations of high intensity and multiple exposure traumas. Also, there is a need develop mental health interventions in Palestine to target individuals who are at risk of suicide. Enhancing mental health services is crucial to mitigate the effects of ongoing traumatic experiences among Palestinians and may lead to a reduction against suicidal ideations and attempts.

## Data Availability

The datasets generated during and/or analyzed during the current study are available from the corresponding author on reasonable request.
